# Casein Kinase 2 Mediates Degradation of Transcription Factor Pcf1 during Appressorium Formation in the Rice Blast Fungus

**DOI:** 10.3390/jof8020144

**Published:** 2022-01-30

**Authors:** Pengyun Huang, Yan Li, Jing Wang, Qing Wang, Zhicheng Huang, Xiaohong Liu, Fucheng Lin, Jianping Lu

**Affiliations:** 1 State Key Laboratory for Managing Biotic and Chemical Threats to the Quality and Safety of Agro-Products, College of Life Sciences, Zhejiang University, Hangzhou 310058, China; huangpengyun2012@163.com (P.H.); LeeYsmailbox@163.com (Y.L.); wj9311@163.com (J.W.); m15760206862_1@163.com (Q.W.); HuangZhiCheng1210@163.com (Z.H.); 2 Institute of Biotechnology, Zhejiang University, Hangzhou 310058, China; xhliu@zju.edu.cn (X.L.); fuchenglin@zju.edu.cn (F.L.); 3 State Key Laboratory for Managing Biotic and Chemical Threats to the Quality and Safety of Agro-Products, Institute of Plant Protection and Microbiology, Zhejiang Academy of Agricultural Sciences, Hangzhou 310021, China

**Keywords:** *Magnaporthe oryzae*, casein kinase 2, transcription factor, protein degradation, appressorium, rice blast

## Abstract

The appressorium is a specialized structure that is differentiated from *Magnaporthe oryzae* spores that can infect host cells. In the process of cellular transformation from spore to appressorium, the contents inside the spores are transferred into appressoria, accompanied by major differences in the gene expression model. In this study, we reported a transcription factor (TF), Pcf1, which was depressed at the transcription level and degraded at the protein level in nuclei of incipient appressoria at four hpi (hours post inoculation). To investigate its degradation mechanism, the interacting proteins of Pcf1 were identified using an immunoprecipitation-mass spectrometry (IP-MS) assay. Yeast two-hybrid (Y2H) and co-IP (co-immunoprecipitation) assays confirmed that Pcf1 interacted with the casein kinase 2 (CK2) holoenzyme through direct combination with the CKb2 subunit. Moreover, Pcf1 was ubiquitinated in the hyphae. These changes in Pcf1 protein levels in nuclei provide a new clue of how TFs are degraded during appressorium formation: temporarily unnecessary TFs in spores are phosphorylated through interacting with CK2 enzyme and are then ubiquitinated and digested by the ubiquitin proteasome system (UPS).

## 1. Introduction

The filamentous fungus *Magnaporthe oryzae* (synonym *Pyricularia oryzae*) causes rice blast disease, which severely threatens global rice production [1]. *M. oryzae* has recently become a model fungus for understanding fungi–plant interactions due to its economic significance and genetic operability [2]. Similar to many plant pathogenic fungi, *M. oryzae* infects its host plant using a highly specialized infection structure that is called an appressorium. To initiate infection, three-celled conidia germinate under the appropriate external environmental conditions, and the tips of germ tubes differentiate to form dome-shaped and melanized appressoria [3]. In this process, the cellular gene expression models of different cell types (conidia and appressoria) are distinct. The unnecessary conidial proteins may be degraded in any of four major pathways: autophagy, ubiquitin proteasome system (UPS), cytosolic proteases, and organelle-mediated degradation systems [4].

Physiological processes, including metabolism, growth, and appressorium formation, in *M. oryzae* are supervised by signaling pathways after receiving environmental signals [5]. Transcriptome analysis shows that genes that are related to protein degradation, such as genes for ubiquitin modification, are dramatically elevated at the beginning of appressorium formation [6]. Recently, the roles and processes of autophagy and UPS during the appressorium formation have been widely reported in *M. oryzae* [7,8,9,10,11,12,13]. UPS is vital for protein degradation and its general process is that after ubiquitin modification, the proteins are degraded through 26S proteasome. The process of ubiquitination is catalyzed by three enzymes: the ubiquitin activating enzyme (E1), the ubiquitin conjugating enzyme (E2), and the ubiquitin ligase (E3) [7]. The impairment of UPS function, through the addition of proteasome inhibitor and the deletion of key genes, inhibits conidial germination and appressorium formation [7,8,9,10]. In addition to the ubiquitination system, autophagy is pivotal in the turnover, trafficking, and transformation of organelles, membranes, and certain proteins, and are eventually vital to cell growth, development, and pathogenicity [11,12,13]. The Δ*atg5* mutant displayed delayed conidial cytoplasm transfer and defects in the transformation of lipid droplets and glycogen into glycerol [14]. The deletion of *ATG14* in *M. oryzae* resulted in impaired conidial autophagic process, delayed breakdown of glycogen, and fewer lipid bodies [15].

Casein kinase 2 (CK2) is a constitutive, conserved, pleiotropic Serine/Threonine kinase in eukaryotic organisms. CK2, which phosphorylates hundreds of substrates, has been documented to be involved in several signaling pathways and is implicated in diverse cellular processes [16,17]. In mammals, CK2 consists of two catalytic subunits (CK2α/α or CK2α/α′) and two regulatory CK2β subunits, and is essential for diverse cellular processes, such as transcription, signaling, proliferation, and cell survival [18,19,20]. In *Saccharomyces cerevisiae*, CK2 holoenzyme, encoded by *CKA1*, *CKA2*, *CKB1*, and *CKB2*, is also heterotrimeric, including two catalytic and two regulatory subunits [21]. CK2 mediates the phosphorylation of Tel2/Tti1, making them the target of degradation by SCFFbxo9 ubiquitin ligase to adjust mTOR signaling to sustain survival in mammals [22]. As a hydrophobic transmembrane protein, hepatitis C virus (HCV) nonstructural 2 (NS2) is phosphorylated by CK2 and then degraded by the proteasome [23]. Phosphorylation that is mediated by CK2 leads to the degradation of the inhibitor of the prosurvival transcription factor (TF) NF-kB (IκBα) through UPS, which is vital for cell survival [24]. Scaglioni et al., reported that the degradation of PML tumor suppressor by UPS shows a CK2-mediated phosphorylation dependency [25]. In *M. oryzae*, CK2 has one catalytic subunit ortholog (CKa1) and two regulatory subunit orthologs (CKb1 and CKb2) [26]. CK2, localized at the nucleus and at the septal and appressorial pores, is responsible for the phosphorylation of intrinsically disordered proteins (IDPs) of septa and nucleoli and is essential for the growth and pathogenicity of *M. oryzae* [26,27]. CK2 is involved in the phosphorylation of Rgs1 (regulator of G-protein signaling) which is localized at the plasma membrane and late endosome, and phosphorylation of Rgs1 is essential for activation of G-protein signaling in *M. oryzae*. Moreover, this interaction between CK2 and Rgs1 was modulated by the endoplasmic reticulum membrane protein complex (EMC) subunit, Emc2 [28]. Transcription factors (TFs) are important links between fungal development and the kinase signaling pathways. Each stage of development, such as growth, conidiogenesis, and appressorium formation, requires spatiotemporally- and elaborately-modulated gene expression, which is vital to the development and pathogenicity of rice blast fungus. Many TFs are involved in the growth of *M. oryzae*, including Tpc1 [29] and Gta1 [30]. Mutants of four zinc TFs (Cos1 [31], Gcc1, Conx1, and Cod1 [30]), homeobox TF Hox2 [32], and three bZIP TFs (Hac1, Bzip10, and MetR) [33] displayed a large reduction or failure of sporulation. By contrast, deleting zinc TF *CNF1* or *CNF2* results in tremendously increased conidiogenesis [30]. Some TFs participate in the regulation of conidial morphology, such as Con7 [34] and Ap1 [35]. Some TFs influence the germination of conidia, such as Tra1 [36] and Mcg1 [37]. Vrf1 selectively regulates appressorium maturation [38]. A MADS-box TF Mcm1 [39] and zinc finger TFs Vrf2, Grf2, and Grf3 [38] are required for the infection process of *M. oryzae*.

All the TFs that are mentioned above regulate one or more processes of development in *M. oryzae*, and their deficiency eventually leads to impaired pathogenicity of *M. oryzae*. Different developmental stages are regulated by different specific TFs. The strict regulation of transcription factor expression and degradation is vital to the appressorium formation process of *M. oryzae*. However, reports on how TFs are degraded between different developmental stages are rare. Here, taking Pcf1 as an example, we studied the degradation of TFs during appressorium differentiation in *M. oryzae*. Our previous study showed that Pcf1 is involved in sporulation, conidial germination, appressorium formation, and virulence in *M. oryzae* [30]. In this study, we further investigated how Pcf1 was degraded in incipient appressoria and tried to learn the degradation process of TFs when conidia germinate to form appressoria. Our study showed that Pcf1 was degraded in the initial stage of appressorium formation and recovered in the late stage. Pcf1 was labeled by ubiquitin and degraded through the UPS, the process of which was mediated by CK2.

## 2. Materials and Methods

### 2.1. Strains and Culture Conditions

All strains that were used in this study were generated from the *M. oryzae* strain 70-15 [40]. Wild-type and other strains were cultured on complete medium (CM) at 25 °C under a 16 h light and 8 h dark phase. Conidia were collected from 9-cm plates regularly and cultured for 12 days followed by centrifugation at 7500 rpm, 5 min after washing with 4 mL distilled water, and filtered using three filter layers. For aerial hyphae collection, 200 μL spore suspension (5 × 10^4^ spores/mL) were inoculated on 9-cm CM plates and cultured for 4 d. For liquid hyphae harvest, about 1 cm^2^ of the hyphae block was first put in a 2 mL tube containing 1 mL CM medium and homogenized using Tissuelyser-24 (Shanghai Jingxin Industrial Development Co., Ltd., Shanghai, China) at 65 Hz for 1 min. The hyphae homogenate was then suspended in 50-100 mL liquid CM medium and shaken at 150 rpm, 25 °C, for 2 d. The hyphae were collected using three layers of filter and washed with water three times. Appressoria were induced on an artificial hydrophobic membrane at 5 × 10^4^–1 × 10^5^ spores/mL concentration at 25 °C. The infected hyphae were collected from 7-day-old barley seedlings that were sprayed with 2 mL 5 × 10^4^ spores/mL conidial suspension of *M. oryzae*.

### 2.2. RNA Isolation and Gene Expression Level Identification

Total RNA at various developmental stages, including aerial hyphae, liquid hyphae, conidia, appressoria, and infectious hyphae was isolated using an RNAiso Plus kit (Takara Biomedical Technology, Beijing, China) [41]. cDNA was synthesized with 500 ng RNA using PrimeScript^TM^ RT reagent Kit (Takara Biomedical Technology, Beijing, China) and then diluted into 50 ng/μL. A 20 μL qRT-PCR mixture was prepared: 10 μL SYBR Premix Ex Taq (Takara Biomedical Technology, Beijing, China), 50 ng cDNA, 1 μL forward primer, 1 μL reverse primer, and 7 μL ddH_2_O. A two-step PCR was performed using an Eppendorf Mastercycler EP Gradient S (Eppendorf, Hamburg, Germany) as follows: 95 °C for 30 s, with 40 cycles at 95 °C for 5 s, and 60 °C for 30 s, followed by a melting curve analysis. The relative abundance of *PCF1* (MGG_17623) in each transcript was calculated using the 2^−∆Ct^ method with *β-TUBULIN* as the reference [42]. The primers for qRT-PCR are listed in supplemental Table S1. The experiment had four biological replicates for each sample.

### 2.3. Subcellular Localization and Fluorescence Intensity Determination

A GFP fusion protein vector, pKD8B-GFP-PCF1, was constructed using a high-throughput gene knockout system as described previously [30]. Briefly, the fragment of *PCF1* coding sequence was amplified from the *M. oryzae* genome was co-transformed into *Saccharomyces cerevisiae* strain FY834 with a linearized pKD8B-GFP that was digested by *Xba*I and *Sal*I [30]. The plasmids of yeast transformants were extracted using TIANprep yeast plasmid DNA kit (Tiangen Biotech, Beijing, China) and transformed into *Escherichia coli* strain DH5α. After confirming the correctness of the vector, pKD8B-GFP-PCF1 and pKD9-H_2_B-mCherry [41] was co-transformed into the wild-type strain through *Agrobacterium tumefaciens*-mediated transformation (ATMT) [30]. Transformants that were expressing both GFP-Pcf1 and H_2_B-mCherry were screened on CM plates that were supplemented with 200 μg/mL hygromycin and 50 mg/mL G418 sulfate. The colocalization of GFP-Pcf1 and H_2_B-mCherry and the fluorescence intensity of GFP-Pcf1 in *M. oryzae* transformants were observed using fluorescence microscopy (Nikon Eclipse Ni) (Nikon, Tokyo, Japan) under the same exposure conditions. The primers for *PCF1* amplification are listed in Supplemental Table S1.

### 2.4. Affinity Purification and Mass Spectrometry Analysis

To identify the proteins that interacted with Pcf1, GFP-Pcf1 with its interacted proteins was immunoprecipitated followed by mass spectrometry. Briefly, 2-day-old hyphae of the transformant expressing *GFP*-*PCF1* were collected and homogenized in protein extraction buffer (50 µM Tris-HCl, 150 mM NaCl, 1 mM EDTA, 1% Triton X-100, pH 7.5, supplemented with 1 mM PMSF and 1× protease inhibitor cocktail (Sigma, Shanghai, China) with a steel ball at 4 °C using Tissuelyser-24 (Shanghai Jingxin industrial development Co., Ltd., Shanghai, China). The hyphae homogenate was centrifuged at 12,000 rpm for 20 min at 4 °C. Then the supernatant was transferred to another tube, mixed with anti-GFP beads (Smart Lifesciences, SA070001, Changzhou, China), and shaken gently for 4 h at 4 °C. The beads were collected by centrifugation at 700× *g* for 2 min and washed once with a low salt washing buffer and once with a high salt washing buffer. The beads were resuspended in 100 µL 0.3 M glycine elution buffer (pH 3.0) and incubated for 15 min with frequent agitation. Finally, the supernatant was pooled into another tube and 10 µL 1 M Tris was added to neutralize the elution buffer. The eluent (10 µL) was used to check whether GFP-Pcf1 was pulled down through IP using anti-GFP antibody (HuaBio, ET1607-31, Hangzhou, China) in a Western blot experiment. Then mass spectrometry of the protein eluent and subsequent comparative analyses were performed as previously described [43].

### 2.5. Yeast Two-Hybrid Assay

The coding region of *PCF1* was amplified from a cDNA library and ligated into *Eco*RI-digested pGBKT7 to construct the bait vector Pcf1-BD; *CKA1*, *CKB1*, and *CKB2* were ligated into *Eco*RI-digested pGADT7 to construct the prey vectors; CKa1-AD, CKb1-AD, and CKb2-AD using ClonExpress^®^ MultiS one step cloning kit (Vazyme, Nanjing, China) following the manufacturer’s instructions. Pairs of bait and prey constructs were transformed into Y2H Golden strain following a small-scale yeast transformation protocol in the pYES2 user manual (Invitrogen, Carlsbad, CA, USA). Separate Pcf1-BD, each with CKa1-AD, CKb1-AD, or CKb2-AD, were used to confirm the interaction between Pcf1 and CK2. pGADT7 with Pcf1-BD and pGBKT7 each with CKa1-AD, CKb1-AD, and CKb2-AD were used to check the self-activation of proteins. pGBKT7-53 with pGADT7-T was used as the positive control, and pGBKT7 with pGADT7 was the negative control. Transformants with different pairs of prey and bait vectors were resuspended in ddH_2_O, diluted into 10^6^, 10^5^, 10^4^, and 10^3^ yeast/mL. The yeast diluent (5 µL) was inoculated on SD-Leu/Trp and SD-Leu/Trp/Ade/His plates and incubated for 4 d at 30 °C. The primers are listed in Table S1. The experiment was repeated three times.

### 2.6. Co-Immunoprecipitation

The full length *CKA1*, *CKB1*, and *CKB2* fragments were amplified using primers that are listed in Table S1, and ligated into the *Xba*I-digested pKD1-3 × FLAG vector using ClonExpress^®^ MultiS one step cloning kit (Vazyme, Nanjing, China). The fusion constructs pKD1-3 × FLAG-CKA1, pKD1-3 × FLAG-CKB1, and pKD1-3 × FLAG-CKB2 were separately transformed into the wild-type strain expressing *GFP-PCF1* through ATMT. Transformants were screened on CM plates that were supplemented with 150 mg/mL glufosinate ammonium. The expression of 3 × FLAG-CKa1, CKb1, and CKb2 in transformants was confirmed using anti-FLAG antibody (HuaBio, M1403-2, Hangzhou, China) in a Western blot assay. The hyphae of the transformants expressing 3 × *FLAG*-*CKA1* and *GFP*-*PCF1*, 3 × *FLAG*-*CKB1,* and *GFP*-*PCF1*, or 3 × *FLAG*-*CKB2* and *GFP-PCF1* were homogenized in protein extraction buffer with a steel ball at 4 °C using Tissuelyser-24 (Shanghai Jingxin Industrial Development Co., Ltd., Shanghai, China). The total protein of each transformant was then separated through centrifugation. Part of the protein extract was set aside as input, and the rest was mixed with anti-FLAG agarose beads (Smart Lifesciences, SA042001, Changzhou, China). After 4 h of gentle agitation, the beads were washed once with a low salt wash buffer and once with a high salt wash buffer. The beads were collected through centrifugation at 700× *g* for 2 min. The proteins that precipitated by anti-FLAG beads were eluted by 100 µL 0.3 M glycine elution buffer (pH 3.0), followed by adding 10 µL 1 M Tris as the neutralizer. Finally, the eluents and input proteins were detected in a Western blot assay with anti-GFP, anti-GAPDH (HuaBio, R1208-3, Hangzhou, China), and anti-FLAG antibodies.

### 2.7. Ubiquitin Detection

The coding sequence of *PCF1* was amplified from the *M. oryzae* genome with primers that are listed in Table S1 and inserted into the *Xba*I-digested pKD1-3 × FLAG vector using ClonExpress^®^ MultiS one step cloning kit (Vazyme, Nanjing, China). pKD1-3 × FLAG-PCF1 vector was transformed into *M. oryzae* through ATMT. After it was screened on the CM medium containing 200 μg/mL hygromycin, the expression of 3 × FLAG-Pcf1 in transformants was detected by a Western blot assay. To immunoprecipitate 3 × FLAG-Pcf1 protein, the 2-day-old hyphae were homogenized in protein extraction buffer with a steel ball at 4 °C. The homogenate was centrifuged at 12,000 rpm for 20 min at 4 °C. The supernatant was then mixed with anti-FLAG Affinity beads (Smart Lifesciences, SA042001, Changzhou, China). After washing with a low salt and a high salt wash buffer, to maximize protein recovery rate, the beads that were combined with 3 × FLAG-Pcf1 were directly boiled in protein loading buffer for 5 min. Then, after centrifuging at 12,000 rpm for 5 min, the 3 × FLAG-Pcf1 protein in the supernatant was detected using anti-ubiquitin antibody (P4D1, sc-8017, Santa Cruz, Shanghai, China) in a Western blot assay.

### 2.8. Statistical Analysis

Data processing in this study was conducted using Tukey’s HSD test in Data Processing System (DPS) [44]. The histograms were displayed as the means ±standard deviation. The different letters represent statistically significant differences (*p* ≤ 0.05).

## 3. Results

### 3.1. Expression of PCF1 Is Repressed in Conidia and Incipient Appressoria

**Figure 1:**
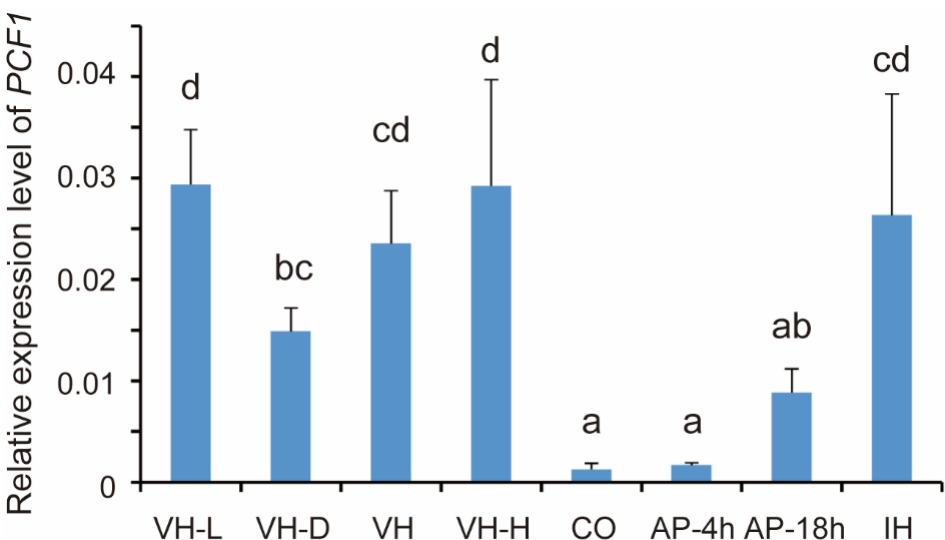
The expression level of *PCF1* at different development stages. VH-L, aerial hyphae in light conditions; VH-D, aerial hyphae in dark conditions; VH, vegetative hyphae; VH-H, vegetative hyphae in starved conditions; CO, conidia; AP-4h, 4 hpi appressoria; AP-18h, 18 hpi appressoria; IH, invasive hyphae. *β-TUBULIN* was used as reference gene. The error bars represent ± SD. Different lowercase letters represent significant differences between the treatments as estimated by Tukey’s HSD test (*p* ≤ 0.05).

The transcription level is an important parameter to estimate whether a gene is required or not in a specific spatiotemporal context. In this study, to evaluate at which stage of development Pcf1 functions, the expression level of *PCF1* was quantified in different development stages. The results showed that the expression of *PCF1* was significantly repressed in conidia and appressorium, compared with aerial, liquid, and invasive hyphae (Figure 1). In particular, *PCF1* was barely expressed in conidia and 4 hpi appressoria. But in 18 hpi appressoria, the expression level of *PCF1* was elevated, even though it was still lower than that in hyphae.

### 3.2. Pcf1 Is Degraded in the Nuclei of Incipient Appressoria

**Figure 2:**
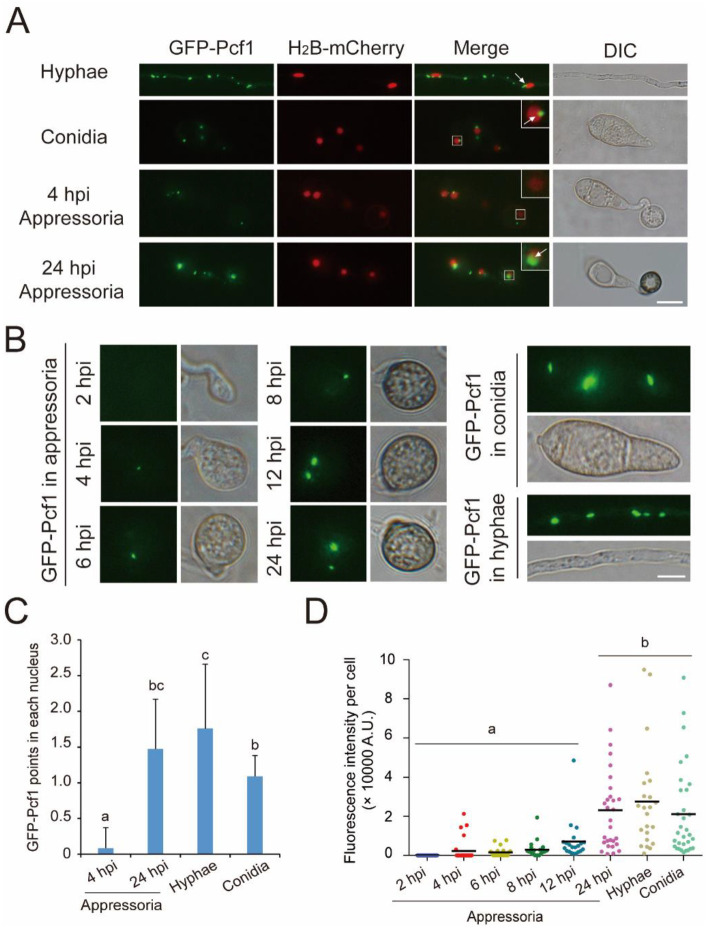
The subcellular localization of Pcf1 and protein intensity of Pcf1 in appressoria. (**A**). Subcellular colocalization of GFP-Pcf1 and H_2_B-mCherry. Bar = 10 μm. (**B**). Fluorescence intensity of GFP-Pcf1 in conidia, hyphae, and at different time points of appressoria. Bar = 5 μm. Arrows point to the margin of a nucleus in which Pcf1 is localized. (**C**). The numbers of GFP-Pcf1 fluorescence points in each nucleus of hyphae, conidia, and 4 and 24 hpi appressoria. The error bars represent ± SD. Different lowercase letters represent significant differences between the cell types as estimated by Tukey’s HSD test (*p* ≤ 0.05). (**D**). Fluorescence intensity of GFP-Pcf1 in a single cell of conidia, hyphae, and appressoria. Different lowercase letters represent significant differences between the cell types as estimated by Tukey’s HSD test (*p* ≤ 0.05). A.U., any unit.

For subcellular localization and the number of fluorescence points, GFP-Pcf1 and H_2_B-mCherry co-localization showed that Pcf1 was distributed in several points and localized in the cytoplasm and partially in the nucleus (Figure 2A). The hyphae contained 5–10 points in each cell, while conidia and appressoria usually only possessed one to three points per cell. In the nuclei, Pcf1 was specifically localized at the margin (Figure 2A). In the nuclei of 4 hpi appressoria, there were no Pcf1-GFP spots, while in the nuclei of hyphae, spores, and 24 hpi appressoria there were one to two Pcf1-GFP spots (Figure 2C).

For the fluorescence intensity of Pcf1-GFP, the hyphae and conidia showed bright fluorescence spots. The Pcf1-GFP fluorescence intensity in appressoria showed an increasing trend from 4 hpi to 24 hpi: 4-hpi appressoria began to show a small Pcf1 point, and 24-hpi appressoria had points with fluorescence intensity that was similar to that in the hyphae and conidia (Figure 2B,D). Combined with the expression level of *PCF1*, the results demonstrated that, in hyphae, Pcf1 existed at a high concentration with high expression; in conidia, Pcf1 maintained a high concentration though without new expression; in appressoria, Pcf1 was degraded in the beginning, then gradually was expressed, and recovered in mature appressoria.

### 3.3. Identification of Proteins Interacting with Pcf1

Pcf1 was degraded when conidia began to form appressoria and then reconstructed in the late period of appressorium formation (Figure 2). To study the degradation mechanism of Pcf1, Pcf1-interacting proteins were identified using immunoprecipitation (IP) that was coupled with mass spectrometry (MS). Pcf1 and its interacting proteins were purified from total protein that was isolated from a wild-type expressing *GFP-PCF1* transformant using anti-GFP beads. Then, the proteins that were interacting with Pcf1 were analyzed by MS. The MS result showed that Pcf1 interacted with nucleus proteins, chaperone proteins, TFs, and kinases (Table 1). Interestingly, three subunits of CK2 holoenzyme (CKa1, CKb1, and CKb2) were all in the reservoir of the interacting protein (Table 1). This result offered a clue that the degradation of Pcf1 was related to CK2.

### 3.4. Pcf1 Interacts with CK2 through CKb2 Subunit

**Figure 3:**
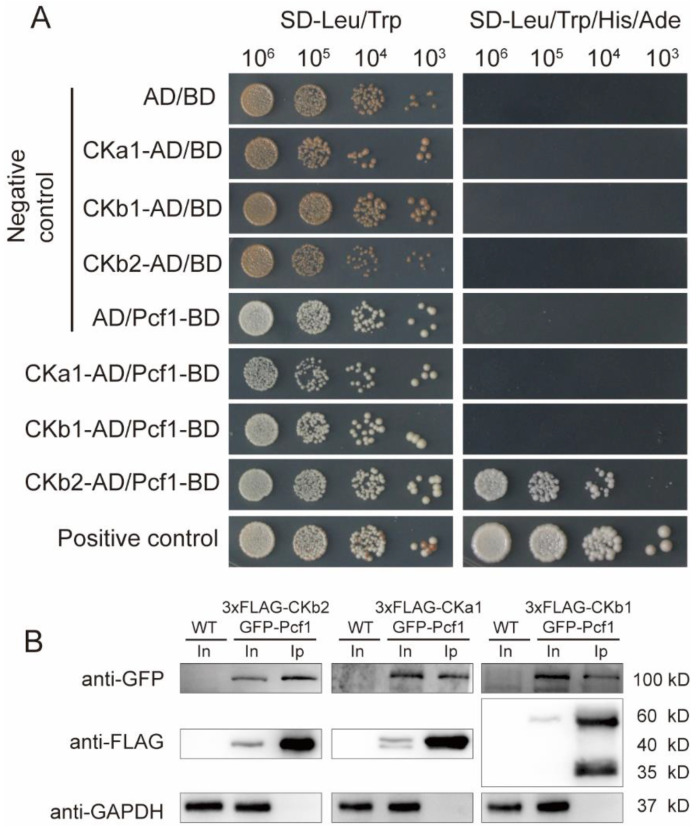
Yeast two-hybrid (Y2H) and co-IP results of CK2 and Pcf1. (**A**). Y2H result between CKa1, Ckb1 and CKb2 subunit and Pcf1. (**B**). Co-IP results between CKa1, CKb1, and CKb2 subunits and Pcf1.

CK2 is a conserved kinase that has various functions in eukaryote cells. In *M. oryzae*, CK2 was involved in appressorium formation and the pathogenic process [26,28]. The direct/indirect and in vitro/in vivo interactions between CK2 and Pcf1 were identified using yeast two-hybrid (Y2H) and co-immunoprecipitation (co-IP) assays. The Y2H results showed that Pcf1 had an intense interaction with the CKb2 subunit in vitro (Figure 3A), because the transformants with CKb2-AD and Pcf1-BD could grow on both SD-Leu/Trp and SD-leu/Trp/His/Ade plates as with the positive control (CKb2-AD/BD and AD/Pcf1-BD were negative controls). Yet Pcf1 did not directly interact with the other two subunits CKa1 and CKb1 (Figure 3A).

In addition to Y2H, co-IP was used to further confirm the interaction between CK2 and Pcf1. The proteins were extracted from the hyphae of strains that were expressing 3 × *FLAG*-*CKA1* and *GFP*-*PCF1*, 3 × *FLAG*-*CKB1* and *GFP*-*PCF1*, or 3 × *FLAG*-*CKB2* and *GFP*-*PCF1* as described above. The proteins were immunoprecipitated using ani-FLAG beads. The eluted proteins from ani-FLAG beads (output sample), total protein (input sample), and proteins that were extracted from wild-type were separated by polyacrylamide gel electrophoresis (Western blot) and detected with anti-FLAG, anti-GFP, and anti-GAPDH antibodies. The results showed that 3 × FLAG-CKa1, CKb1, andCKb2 all precipitated with GFP-Pcf1, as GFP-Pcf1 was detected in the elution of the respective co-IPs (Figure 3B). This result indicated that Pcf1 interacted with CK2 holoenzyme in vivo. Therefore, Pcf1 interacts with CK2 holoenzyme directly through interacting with CKb2 subunit.

### 3.5. Pcf1 Is Ubiquitinated in the Hyphae

**Figure 4:**
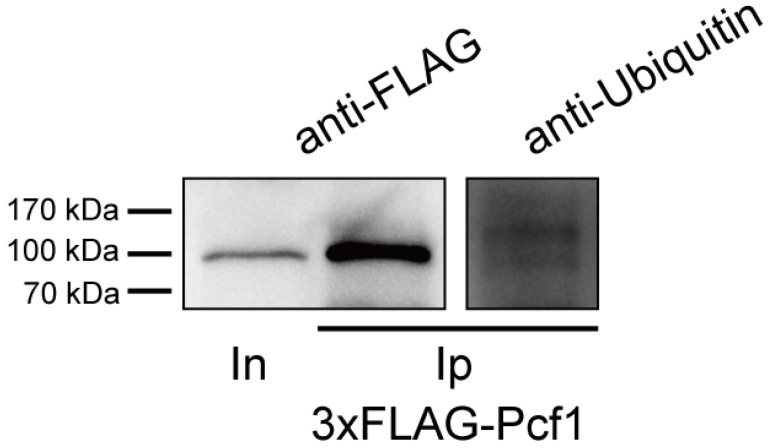
The ubiquitin detection of Pcf1. Pcf1 was extracted by anti-FLAG beads and then detected using anti-ubiquitin antibody.

The UPS is responsible for the degradation of more than 80% of intracellular proteins to maintain metabolic homeostasis [49]. Ubiquitination is catalyzed to link target protein by E1 ubiquitin-activating enzyme, E2 ubiquitin-conjugating enzyme, and E3 ubiquitin-ligase. The most ubiquitinated substrate is recognized and degraded by 26S proteasome [50]. To learn the underlying mechanism of Pcf1 degradation, 3 × FLAG-Pcf1 was immunoprecipitated from wild-type expressing 3 × *FLAG-PCF1* transformant by anti-FLAG beads and used to detect ubiquitination of Pcf1, with the detection of 3 × FLAG-Pcf1 as the control. The results showed that a small part of Pcf1 was labeled by ubiquitin, with a relatively weak band that was larger than 3 × FLAG-Pcf1, because the molecular weight of Pcf1 protein was increased after ubiquitination (Figure 4). The ubiquitination of 3 × FLAG-Pcf1 demonstrated that Pcf1 may be digested through the UPS.

## 4. Discussion

As highly specialized infection structures, appressoria experience complex cellular changes during their development process from conidia. Unnecessarily old proteins that are specific to spores are degraded, while new proteins that are specific to appressoria are synthesized again. In this study, we demonstrated that, in the early stage of appressorium formation, the TF Pcf1, which is required for fungal development and virulence in *M. oryzae*, is degraded through the UPS that is mediated by the CK2 holoenzyme in appressorial nuclei.

In response to environmental signals, such as temperature, humidity, hydrophobicity, or nutrients, the appressorium development process in *M. oryzae* is strictly and spatiotemporally regulated by signaling pathways (such as cAMP/PKA and MST11-MST7-PMK1 MAPK) and is eventually executed through the expression of related specific genes [51,52,53]. Transcription factors are important links in the appressorium formation process from sensing induction signals to the expression of specific genes through the kinase signaling pathway. Some TFs have been shown to specifically regulate the expression of genes that are essential to different stages of development in *M. oryzae*. For example, Cos1 specifically regulates sporulation of *M. oryzae* without influencing growth [54]. Hox7 and Vrf1 specifically participate in the development of appressoria, without influencing vegetative growth [32,38]. Park et al., studied the expression patterns of 206 TF genes in *M. oryzae* under 32 conditions, and found that 31 TFs, such as *HOX7* and *CRF1*, were upregulated in all infection-related stages, including conidiation, germination, and appressorium formation. Other homeobox TFs (*HOX1*, *HOX2*, *HOX3*, *HOX4*, *HOX6*, and *HOX8*), *COS1*, *CON7*, *NIT4*, and *ACR1* were all upregulated during conidiation [55]. In contrast, other TFs function as repressors to inhibit the expression of appressorium-specific genes in the developmental stages other than appressorium, which need to be degraded in the appressorium formation process. By chance, we found that Pcf1 was inhibited at the transcriptional level in conidia and appressoria (Figure 1). Based on the low transcription level, conidia and the early stages of appressoria do not synthesize more Pcf1 proteins. In addition, Pcf1 was degraded in incipient appressoria at the protein level, yet elevated in the mature appressoria at both the transcription and protein levels (Figure 2). This result indicated that Pcf1 was not necessary for the initial stage of appressorium formation, but functioned in hyphae, conidia, and mature appressoria.

Casein kinase 2 (CK2), a heterotrimeric holoenzyme, is a ubiquitous and conserved kinase in eukaryotic organisms. In mammals, CK2 is required for the phosphorylation and degradation of substrate proteins [22,23,24]. In *M. oryzae*, one catalytic subunit ortholog (CKa1) and two regulatory subunit orthologs (CKb1 and CKb2) have been identified through homology comparison [26]. CK2 is located at the nucleus and at septal and appressorial pores and has been demonstrated to be involved in phosphorylating intrinsically disordered proteins [26,27]. Coincidently, Pcf1 has a similar distribution pattern to CK2. Additionally, in *M. oryzae*, modulated by the EMC subunit (Emc2), CK2 is responsible for the phosphorylation of Rgs1 [28]. Our results demonstrated that Pcf1 interacts with CK2 both in vitro and in vivo. The co-IP result in Figure 3B confirmed that Pcf1 is interacted with Ck2 holoenzyme in vivo due to co-precipitation with all three subunits. The in vitro Y2H result indicated that Pcf1 is directly interacting with the CKb2 subunit (Figure 3A). Here, from the interaction relationship with Pcf1 and Ck2, we reasoned that Pcf1 is phosphorylated by CK2.

The ubiquitin proteasome system is responsible for, but not limited to, protein degradation to maintain protein homeostasis [49]. Ubiquitination of substrate proteins is implemented by a sequential cascade of three enzymes: E1, E2, and E3. In *M. oryzae*, the impairment of UPS function, including the addition of a proteasome inhibitor [7], and the deletion of ubiquitin ligase genes *FWD1* (a gene of F-box protein) [8] and *UBR1* (a gene encoding ubiquitin ligases) [9] all resulted in defects in conidial germination and appressorium formation, indicating that the UPS is essential for initiating appressorium development. Moreover, ubiquitination is also critical in rice immunity during plant–pathogen interaction [56,57]. Our results showed that Pcf1 is modified by ubiquitin, suggesting that Pcf1 is degraded through UPS. However, more research is needed to provide more evidence in the further.

In summary, the process of appressorium formation is accompanied by tremendous changes of substance in conidia. Pcf1 is a transcription factor that is depressed/degraded and recovered during the initial stage and the late stage of appressorium formation, respectively. As such, taking the transcription factor Pcf1 as the representative protein, we demonstrated that unnecessarily old proteins in conidia are degraded through the ubiquitin proteasome system after phosphorylation by CK2 in the initial stage of appressorium development in *M. oryzae*.

## Figures and Tables

**Table 1:** Putative Pcf1-interacting proteins that were identified by IP-MS in *M. oryzae*.

Protein	Locus	Description	% PSMs *	Reference
CKa1/CK2a	MGG_03696	Casein kinase II subunit alpha	23	[26]
CKb1/CK2b1	MGG_00446	Casein kinase II subunit beta-1	19	[26]
CKb2/CK2b2	MGG_05651	Casein kinase II subunit beta-2	1	[26]
Yck1	MGG_02829	Casein kinase I	6	[2]
Rad3	MGG_12633	The ortholog of protein kinase Rad3 in *S. pombe*	3	
Kin1	MGG_01279	Camkl Kin1 protein kinase	1	[45]
Hik3	MGG_12530	Histidine kinase	1	[46]
Hik1	MGG_11174	Histidine kinase	1	[46]
Sik1	MGG_07915	Pre-rRNA processing nucleolar protein Sik1	7	
Nop58	MGG_07008	Nucleolar protein Nop-58	5	
Ssr4	MGG_00174	SWI/SNF and RSC complexes subunit Ssr4	2	
Swi3	MGG_01720	The SWI/SNF chromatin remodeling complex	4	
Nop2	MGG_01292	Nucleolar protein Nop2	2	[47]
Arp8	MGG_05229	INO80 chromatin remodeling complex subunit (Arp8)	1	
Ies1	MGG_08312	INO80 chromatin remodeling complex	1	
RuvB-like helicase 1	MGG_03958	INO80 chromatin remodeling complex	2	
Fzc53	MGG_09829	Zn(2)-Cys(6) zinc finger domain protein	1	[30]
Tup1	(MGG_08829)	Transcriptional repressor Rco-1	1	[48]

* PSMs: peptide spectrum matches.

## Data Availability

The data presented in this study are available in this published article or its supplementary materials.

## Supplementary Materials

The following are available online at https://www.mdpi.com/article/10.3390/jof8020144/s1, Table S1: Primers used in this study.

## References

[1] Ryder L.S., Dagdas Y.F., Kershaw M.J., Venkataraman C., Madzvamuse A., Yan X., Cruz-Mireles N., Soanes D.M., Oses-Ruiz M., Styles V. (2019). A sensor kinase controls turgor-driven plant infection by the rice blast fungus. Nature.

[2] Shi H., Chen N., Zhu X., Su Z., Wang J., Lu J., Liu X., Lin F. (2019). The casein kinase MoYck1 regulates development, autophagy, and virulence in the rice blast fungus. Virulence.

[3] Cooper J., Donofrio N. (2021). Regulators unite to enable plant entry. Nat. Microbiol..

[4] Liu X., Ning G., Huang L., Zhao Y., Dong B., Lu J., Lin F. (2016). Calpains are involved in asexual and sexual development, cell wall integrity and pathogenicity of the rice blast fungus. Sci. Rep..

[5] He M., Xu Y., Chen J., Luo Y., Lv Y., Su J., Kershaw M.J., Li W., Wang J., Yin J. (2018). MoSnt2-dependent deacetylation of histone H3 mediates MoTor-dependent autophagy and plant infection by the rice blast fungus *Magnaporthe oryzae*. Autophagy.

[6] Oh Y., Donofrio N., Pan H., Coughlan S., Brown D.E., Meng S., Mitchell T., Dean R.A. (2008). Transcriptome analysis reveals new insight into appressorium formation and function in the rice blast fungus *Magnaporthe oryzae*. Genome Biol..

[7] Oh Y., Franck W.L., Han S.O., Shows A., Gokce E., Muddiman D.C., Dean R.A. (2012). Polyubiquitin is required for growth, development and pathogenicity in the rice blast fungus *Magnaporthe oryzae*. PLoS ONE.

[8] Shi H., Chen N., Zhu X., Liang S., Li L., Wang J., Lu J., Lin F., Liu X. (2019). F-box proteins MoFwd1, MoCdc4 and MoFbx15 regulate development and pathogenicity in the rice blast fungus *Magnaporthe oryzae*. Environ. Microbiol..

[9] Shi H., Chen G., Chen Y., Dong B., Lu J., Liu X., Lin F. (2016). MoRad6-mediated ubiquitination pathways are essential for development and pathogenicity in *Magnaporthe oryzae*. Environ. Microbiol..

[10] Wang Y., Yang N., Zheng Y., Yue J., Bhadauria V., Peng Y., Chen Q. (2022). Ubiquitination in the rice blast fungus *Magnaporthe oryzae*: From development and pathogenicity to stress responses. Phytopathol. Res..

[11] Zheng W., Zhou J., He Y., Xie Q., Chen A., Zheng H., Shi L., Zhao X., Zhang C., Huang Q. (2015). Retromer is essential for autophagy-dependent plant infection by the rice blast fungus. PLoS Genet..

[12] Deng Y., Ramos-Pamplona M., Naqvi N.I. (2009). Autophagy-assisted glycogen catabolism regulates asexual differentiation in *Magnaporthe oryzae*. Autophagy.

[13] Zhu X., Li L., Wu M., Liang S., Shi H., Liu X., Lin F. (2019). Current opinions on autophagy in pathogenicity of fungi. Virulence.

[14] Lu J., Liu X., Feng X., Min H., Lin F. (2009). An autophagy gene, MgATG5, is required for cell differentiation and pathogenesis in *Magnaporthe oryzae*. Curr. Genet..

[15] Liu X., Zhao Y., Zhu X., Zeng X., Huang L., Dong B., Su Z., Wang Y., Lu J., Lin F. (2017). Autophagy-related protein MoAtg14 is involved in differentiation, development and pathogenicity in the rice blast fungus *Magnaporthe oryzae*. Sci. Rep..

[16] Borgo C., D’Amore C., Sarno S., Salvi M., Ruzzene M. (2021). Protein kinase CK2: A potential therapeutic target for diverse human diseases. Signal Transduct. Target. Ther..

[17] Masłyk M., Kochanowicz E., Zieliński R., Kubiński K., Hellman U., Szyszka R. (2008). Yeast surviving factor Svf1 as a new interacting partner, regulator and in vitro substrate of protein kinase CK2. Mol. Cell. Biochem..

[18] Filhol O., Giacosa S., Wallez Y., Cochet C. (2015). Protein kinase CK2 in breast cancer: The CK2β regulatory subunit takes center stage in epithelial plasticity. Cell. Mol. Life Sci..

[19] Guerra B., Issinger O.G. (1999). Protein kinase CK2 and its role in cellular proliferation, development and pathology. Electrophoresis.

[20] Unger G.M., Davis A.T., Slaton J.W., Ahmed K. (2004). Protein kinase CK2 as regulator of cell survival: Implications for cancer therapy. Curr. Cancer Drug Targets.

[21] Glover C.V., Bidwai A.P., Reed J.C. (1994). Structure and function of *Saccharomyces cerevisiae* casein kinase II. Mol. Cell. Biol..

[22] Fernández-Sáiz V., Targosz B.-S., Lemeer S., Eichner R., Langer C., Bullinger L., Reiter C., Slotta-Huspenina J., Schroeder S., Knorn A.-M. (2013). SCFFbxo9 and CK2 direct the cellular response to growth factor withdrawal via Tel2/Tti1 degradation and promote survival in multiple myeloma. Nat. Cell Biol..

[23] Franck N., Le Seyec J., Guguen-Guillouzo C., Erdtmann L. (2005). Hepatitis C virus NS2 protein is phosphorylated by the protein kinase CK2 and targeted for degradation to the proteasome. J. Virol..

[24] Kato T., Delhase M., Hoffmann A., Karin M. (2003). CK2 Is a C-Terminal IkappaB Kinase Responsible for NF-kappaB Activation during the UV Response. Mol. Cell.

[25] Scaglioni P.P., Yung T.M., Choi S.C., Baldini C., Konstantinidou G., Pandolfi P.P. (2008). CK2 mediates phosphorylation and ubiquitin-mediated degradation of the PML tumor suppressor. Mol. Cell. Biochem..

[26] Zhang L., Zhang D., Chen Y., Ye W., Lin Q., Lu G., Ebbole D.J., Olsson S., Wang Z. (2019). *Magnaporthe oryzae* CK2 accumulates in nuclei, nucleoli, at septal pores and forms a large ring structure in appressoria, and is involved in rice blast pathogenesis. Front. Cell. Infect. Microbiol..

[27] Zhang L., Zhang D., Liu D., Li Y., Li H., Xie Y., Wang Z., Hansen B.O., Olsson S. (2020). Conserved eukaryotic kinase CK2 chaperone intrinsically disordered protein interactions. Appl. Environ. Microbiol..

[28] Yu R., Shen X., Liu M., Liu X., Yin Z., Li X., Feng W., Hu J., Zhang H., Zheng X. (2021). The rice blast fungus MoRgs1 functioning in cAMP signaling and pathogenicity is regulated by casein kinase MoCk2 phosphorylation and modulated by membrane protein MoEmc2. PLoS Pathog..

[29] Galhano R., Illana A., Ryder L.S., Rodríguez-Romero J., Demuez M., Badaruddin M., Martinez-Rocha A.L., Soanes D.M., Studholme D.J., Talbot N.J. (2017). Tpc1 is an important Zn(II)2Cys6 transcriptional regulator required for polarized growth and virulence in the rice blast fungus. PLoS Pathog..

[30] Lu J., Cao H., Zhang L., Huang P., Lin F. (2014). Systematic analysis of Zn2Cys6 transcription factors required for development and pathogenicity by high-throughput gene knockout in the rice blast fungus. PLoS Pathog..

[31] Li X., Han X., Liu Z., He C. (2013). The function and properties of the transcriptional regulator COS1 in *Magnaporthe oryzae*. Fungal. Biol..

[32] Kim S., Park S.Y., Kim K.S., Rho H.S., Chi M.H., Choi J., Park J., Kong S., Park J., Goh J. (2009). Homeobox transcription factors are required for conidiation and appressorium development in the rice blast fungus *Magnaporthe oryzae*. PLoS Genet..

[33] Tang W., Ru Y., Hong L., Zhu Q., Zuo R., Guo X., Wang J., Zhang H., Zheng X., Wang P. (2015). System-wide characterization of bZIP transcription factor proteins involved in infection-related morphogenesis of *Magnaporthe oryzae*. Environ. Microbiol..

[34] Odenbach D., Breth B., Thines E., Weber R.W., Anke H., Foster A.J. (2007). The transcription factor Con7p is a central regulator of infection-related morphogenesis in the rice blast fungus Magnaporthe grisea. Mol. Microbiol..

[35] Guo M., Chen Y., Du Y., Dong Y., Guo W., Zhai S., Zhang H., Dong S., Zhang Z., Wang Y. (2011). The bZIP transcription factor MoAP1 mediates the oxidative stress response and is critical for pathogenicity of the rice blast fungus *Magnaporthe oryzae*. PLoS Pathog..

[36] Breth B., Odenbach D., Yemelin A., Schlinck N., Schröder M., Bode M., Antelo L., Andresen K., Thines E., Foster A.J. (2013). The role of the Tra1p transcription factor of *Magnaporthe oryzae* in spore adhesion and pathogenic development. Fungal. Genet. Biol..

[37] Li H., Lu J., Khan I.A., Zhang L., He R., Lin F. (2011). A novel *Magnaporthe oryzae* gene MCG1, encoding an extracellular globular protein, affects conidial germination and appressorial formation. Int. J. Agric. Technol..

[38] Cao H., Huang P., Zhang L., Shi Y., Sun D., Yan Y., Liu X., Dong B., Chen G., Snyder J.H. (2016). Characterization of 47 Cys2 -His2 zinc finger proteins required for the development and pathogenicity of the rice blast fungus *Magnaporthe oryzae*. New Phytol..

[39] Zhou X., Liu W., Wang C., Xu Q., Wang Y., Ding S., Xu J. (2011). A MADS-box transcription factor MoMcm1 is required for male fertility, microconidium production and virulence in *Magnaporthe oryzae*. Mol. Microbiol..

[40] Chao C.T., Ellingboe A.H. (1991). Selection for mating competence in *Magnaporthe grisea* pathogenic to rice. Can. J. Bot..

[41] Sun D., Cao H., Shi Y., Huang P., Dong B., Liu X., Lin F., Lu J. (2017). The regulatory factor X protein MoRfx1 is required for development and pathogenicity in the rice blast fungus *Magnaporthe oryzae*. Mol. Plant Pathol..

[42] Schmittgen T.D., Livak K.J. (2008). Analyzing real-time PCR data by the comparative C(T) method. Nat. Protoc..

[43] Zhu X., Li L., Cai Y., Wu X., Shi H., Liang S., Qu Y., Naqvi N.I., Del Poeta M., Dong B. (2021). A VASt-domain protein regulates autophagy, membrane tension, and sterol homeostasis in rice blast fungus. Autophagy.

[44] Tang Q., Zhang C. (2013). Data Processing System (DPS) software with experimental design, statistical analysis and data mining developed for use in entomological research. Insect Sci..

[45] Luo Y., Zhang H., Qi L., Zhang S., Zhou X., Zhang Y., Xu J. (2014). FgKin1 kinase localizes to the septal pore and plays a role in hyphal growth, ascospore germination, pathogenesis, and localization of Tub1 beta-tubulins in *Fusarium graminearum*. New Phytol..

[46] Jacob S., Foster A.J., Yemelin A., Thines E. (2014). Histidine kinases mediate differentiation, stress response, and pathogenicity in *Magnaporthe oryzae*. MicrobiologyOpen.

[47] Pavlopoulou A., Kossida S. (2009). Phylogenetic analysis of the eukaryotic RNA (cytosine-5)-methyltransferases. Genomics.

[48] Chen Y., Zhai S., Sun Y., Li M., Dong Y., Wang X., Zhang H., Zheng X., Wang P., Zhang Z. (2015). MoTup1 is required for growth, conidiogenesis and pathogenicity of *Magnaporthe oryzae*. Mol. Plant Pathol..

[49] Chen Y., Wu H., Shen X. (2016). The ubiquitin-proteasome system and its potential application in hepatocellular carcinoma therapy. Cancer Lett..

[50] Qi S., Dong J., Xu Z., Cheng X., Zhang W., Qin J. (2021). PROTAC: An effective targeted protein degradation strategy for cancer therapy. Front. Pharmacol..

[51] Anjago W.M., Zhou T., Zhang H., Shi M., Yang T., Zheng H., Wang Z. (2018). Regulatory network of genes associated with stimuli sensing, signal transduction and physiological transformation of appressorium in *Magnaporthe oryzae*. Mycology.

[52] Selvaraj P., Shen Q., Yang F., Naqvi N.I. (2017). Cpk2, a catalytic subunit of cyclic AMP-PKA, Regulates growth and pathogenesis in rice blast. Front. Microbiol..

[53] Turrà D., Segorbe D., Di Pietro A. (2014). Protein kinases in plant-pathogenic fungi: Conserved regulators of infection. Annu. Rev. Phytopathol..

[54] He N., An B., Xie Q., Yan X., Feng H., Luo H., He C. (2019). Synergistic deletion of RGS1 and COS1 may reduce the pathogenicity of *Magnaporthe oryzae*. Arch. Microbiol..

[55] Park S.Y., Choi J., Lim S.E., Lee G.W., Park J., Kim Y., Kong S., Kim S.R., Rho H.S., Jeon J. (2013). Global expression profiling of transcription factor genes provides new insights into pathogenicity and stress responses in the rice blast fungus. PLoS Pathog..

[56] Park C.H., Chen S., Shirsekar G., Zhou B., Khang C.H., Songkumarn P., Afzal A.J., Ning Y., Wang R., Bellizzi M. (2012). The *Magnaporthe oryzae* effector AvrPiz-t targets the RING E3 ubiquitin ligase APIP6 to suppress pathogen-associated molecular pattern-triggered immunity in rice. Plant Cell.

[57] Liu X., Song L., Zhang H., Lin Y., Shen X., Guo J., Su M., Shi G., Wang Z., Lu G. (2021). Rice ubiquitin-conjugating enzyme OsUBC26 is essential for immunity to the blast fungus *Magnaporthe oryzae*. Mol. Plant Pathol..

