# Supplementary material for: Casein Kinase 2 Mediates Degradation of Transcription Factor Pcf1 during Appressorium Formation in the Rice Blast Fungus
Source: J Fungi (Basel). 2022 Jan 30;8(2):144. doi: 10.3390/jof8020144 (PMC8878131; doi:10.3390/jof8020144)
Supplement: Supplementary file 1 [file jof-08-00144-s001.zip › jof-1565943-supplementary.pdf]

Table S1. Primers used in this study

Primer	Sequence
**pKD8-GFP-Pcf1-F**	ATCACTCTCGGCATGGACGAGCTGTACAAGGGATCCACGCAGGATACTGTAGAGTCCGG
**pKD8-GFP-Pcf1-R**	CGTATCGTGATGGCGTGTCGCTTACTGCAGGTCGACCACAATAGCCCAATCCCGACTGA
**Pcf1-qrt-F**	GAATGGGTGTTTGACTTGCAG
**Pcf1-qrt-R**	GCATCGGAGCTTGTGGTAG
**Cka1-ADF**	GCCATGGAGGCCAGTGAATTCATGCACAGCATGGCGCGCGTT
**Cka1-ADR**	ATGCCCTCCCGGGTGGAATTCTCAAGCCGAGGTGTTGGTTCC
**Ckb1-ADF**	GCCATGGAGGCCAGTGAATTCATGTCGACTTCGTCGGGAACG
**Ckb1-ADR**	ATGCCCTCCCGGGTGGAATTCTCACAATTCCGACTCTGCCCC
**Ckb2-ADF**	GCCATGGAGGCCAGTGAATTCATGGAAGACTTTGGCAGCGAG
**Ckb2-ADR**	ATGCCCTCCCGGGTGGAATTCTCAGACACCTTGCATCATGCT
**Pcf1-BDF**	ATGGCCATGGAGGCCGAATTCATGACGCAGGATACTGTAGAG
**Pcf1-BDR**	TCGACGGATCCCCGGGAATTCTTACACAATAGCCCAATCCCG
**3flag-Cka1F**	GACGATGATGACAAGTCTAGAATGCACAGCATGGCGCGCGTT
**3flag-Cka1R**	TTACTGCAGGTCGACTCTAGATCAAGCCGAGGTGTTGGTTCC
**3flag-Ckb1F**	GACGATGATGACAAGTCTAGAATGTCGACTTCGTCGGGAACG
**3flag-Ckb1R**	TTACTGCAGGTCGACTCTAGATCACAATTCCGACTCTGCCCC
**3flag-Ckb2F**	GACGATGATGACAAGTCTAGAATGGAAGACTTTGGCAGCGAG
**3flag-Ckb2R**	TTACTGCAGGTCGACTCTAGATCAGACACCTTGCATCATGCT
**3flag-Pcf1F**	GACGATGATGACAAGTCTAGAATGACGCAGGATACTGTAGAG
**3flag-Pcf1R**	TTACTGCAGGTCGACTCTAGATTACACAATAGCCCAATCCCG
